# Learning from single timestamps: complexity estimation in laparoscopic cholecystectomy

**DOI:** 10.1007/s11548-026-03648-6

**Published:** 2026-05-19

**Authors:** Dimitrios Anastasiou, Santiago Barbarisi, Lucy Culshaw, Jayna Patel, Evangelos B. Mazomenos, Imanol Luengo, Danail Stoyanov

**Affiliations:** 1 UCL Hawkes Institute, University College London, London, UK; 2 Medtronic plc., London, UK; 3 Department of Medical Physics and Biomedical Engineering, University College London, London, UK; 4 Department of Computer Science, University College London, London, UK

**Keywords:** Surgical complexity, Laparoscopic cholecystectomy, Parkland Grading Scale, Temporal localization, Weak supervision, Surgical data science

## Abstract

**Purpose:**

Accurate assessment of surgical complexity is essential in laparoscopic cholecystectomy (LC), where severe inflammation is associated with longer operative times and increased risk of postoperative complications. The Parkland Grading Scale (PGS) provides a clinically validated framework for stratifying inflammation severity; however, its automation in surgical videos remains largely unexplored, particularly in realistic scenarios where complete videos must be analyzed without prior manual curation.

**Methods:**

In this work, we introduce STC-Net, a novel framework for Single-Timestamp-based Complexity estimation in LC via the PGS, designed to operate under weak temporal supervision. Unlike prior methods limited to static images or manually trimmed clips, STC-Net operates directly on full videos. It jointly performs temporal localization and grading through a localization, window proposal, and grading module. We introduce a novel loss formulation combining hard and soft localization objectives and background-aware grading supervision.

**Results:**

Evaluated on a private dataset of 1859 LC videos, STC-Net achieves an accuracy of 60.18% and an F1-score of 59.68%, outperforming non-localized baselines by over 12% in both metrics and highlighting the effectiveness of weak supervision for surgical complexity assessment.

**Conclusion:**

STC-Net demonstrates a scalable and effective approach for automated PGS-based surgical complexity estimation from full LC videos, making it promising for postoperative analysis and surgical training.

**Supplementary Information:**

The online version contains supplementary material available at 10.1007/s11548-026-03648-6.

## Introduction

Laparoscopic cholecystectomy (LC) is one of the most common surgical procedures for the treatment of cholecystitis [1]. Yet, its intraoperative course can vary significantly due to the wide spectrum of underlying pathologies, ranging from mild biliary colic to gangrenous cholecystitis [2]. Severe inflammation is associated with greater technical difficulty, longer operative times, and increased risk of complications [3].

**Fig. 1:**
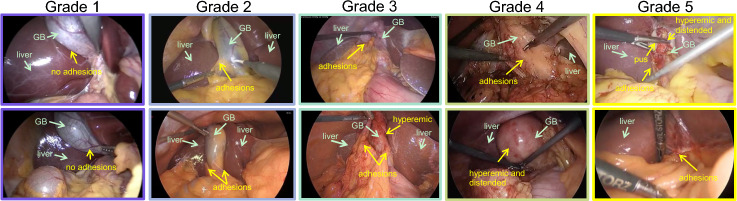
Examples of different PGS grades from our dataset. The screenshots correspond to the moment at which the PGS grade was assigned. GB stands for gallbladder

Given the variability of gallbladder inflammation, reliable grading systems are essential for stratifying LC complexity. Such systems can inform intraoperative decision-making [4] and enable case stratification to support surgical training and education. Among those, such as G10, Nassar, and the Parkland Grading Scale (PGS) [4–6], the PGS is widely adopted for its objective criteria and strong inter-rater reliability [2, 6]. It is a 5-point scale assigned early in the operation based on visual and anatomical features such as adhesions, distension, and necrosis [6]. Higher PGS grades correlate with greater operative difficulty and the risk of complications, underscoring the need for an accurate assessment [7]. Representative examples of PGS grades are shown in Fig. 1 (see Online Resource 1 for detailed descriptions).

The growing availability of surgical video has enabled the automation of many applications in LC, such as workflow recognition, CVS detection, and anatomy segmentation [8–13]. Building on these advances, automating PGS estimation offers a standardized alternative to subjective intraoperative assessment by surgeons, thus providing a reproducible measure of surgical complexity.

Automated PGS estimation aims to predict the grade of gallbladder inflammation severity from LC videos. This grade is typically assigned during the initial inspection phase of the gallbladder. To support model training, two types of annotations are required: (i) the PGS label and (ii) the temporal window (start and end timestamps) that defines the grading period. In real clinical settings, the model must estimate the PGS grade directly from raw, full-length videos without relying on manually trimmed clips (i.e., clip relevant to grading as in [14]). Manual trimming is time-consuming, subjective, and not available at scale, making full-length inference essential for real-world deployment. However, naively grading the entire full-length video is ineffective due to long irrelevant segments, motivating an explicit localization mechanism. This requires the model to learn how to localize the relevant segment for grading during training. While providing full temporal annotations (i.e., start and end timestamps) can facilitate model training, such annotations are time-consuming to generate and often ambiguous. A more practical alternative is to annotate a single timestamp that marks a representative moment for grading, which avoids the need to define and refine start/end boundaries for a region that is often not clearly defined in time. In our dataset, these timestamps predominantly occur early in the operation (see Online Resource 1), consistent with the initial inspection phase. This form of weak temporal supervision can effectively guide the model in localizing informative segments for PGS estimation, offering a middle ground between fully supervised and fully unlocalized (no temporal annotations) approaches.

In this work, we propose **STC-Net**, a novel framework for **S**ingle-**T**imestamp-based **C**omplexity estimation in LC using the PGS. To the best of our knowledge, STC-Net is the first method to estimate PGS directly from full-length LC videos without manual trimming at test time, making it suitable for real postoperative use. Trained under weak temporal supervision [15, 16], where each video is labeled with a single timestamp and its PGS grade, STC-Net jointly localizes informative segments and predicts the PGS grade. It comprises three components: a *Localization Module* (LM) that assigns frame-wise relevance scores, a *Window Proposal Module* (WPM) that extracts candidate temporal segments based on localization confidence, and a *Grading Module* (GM) that classifies segments and aggregates PGS predictions via a consensus strategy. A novel supervision scheme further enforces synergy between localization and grading objectives. Our main contributions are:

- We propose STC-Net, the first framework to estimate PGS in LC directly from full-length surgical videos, thereby supporting realistic postoperative workflows without manual trimming.
- We design the WPM that generates adaptive, video-specific temporal segments around predicted timestamps (via Gaussian-based window slicing), improving the relevance of inputs to the grading model.
- We introduce a novel training strategy that couples localization and grading objectives, including (i) a two-part localization loss combining hard (binary) and soft (distributional) supervision and (ii) a background-aware grading loss that explicitly models non-informative proposals to reduce noisy supervision.
- We evaluate STC-Net through extensive experiments on a large private dataset of LC procedures.

**Related work:** Existing works that address PGS (or other grading scale) estimation in LC videos often simplify the task by framing it as frame-wise classification, training and evaluating models on isolated static images. Abbing et al. [17] trained convolutional neural networks (CNNs) on still images labeled with a modified Nassar score. Wu et al. [18] proposed a framework for surgical phase recognition, CVS detection and PGS estimation, and Ward et al. [2] used CNNs to classify PGS grades from static images. While these approaches may perform well on curated images, they do not reflect the real-world scenario, where the specific frames relevant to grading are unknown at test time. Moreover, inflammation severity may not be evident in still frames, requiring temporal cues such as tissue manipulation or bleeding for accurate assessment. A more temporally oriented approach was proposed by Ban et al. [14], trained and evaluated on 16-second video clips capturing the initial inspection of the gallbladder. It uses a concept graph neural network to model surgical concepts associated with inflammation. However, despite leveraging temporal information, their setup still assumes prior knowledge of when grading occurs (testing on trimmed clips).

## Methods

For a surgical video with *T* frames, we pre-compute frame-level representations, resulting in features $X \in \mathbb {R}^{T\times D}$, where *D* is the feature dimension. Each video is annotated with a PGS class label denoted by $c \in \{1, \dots , C\}$, where *C* is the number of PGS classes, and a target timestamp $t \in \mathbb {N}$ marking the temporal location where the grade was assigned. The timestamp can be converted into a one-hot vector $y \in \{0,1\}^T$ as $y_i = 1$ if i=t, and $y_i = 0$ otherwise, for i=0,⋯,T-1. This forms the basis of our weakly supervised setting, where the model is trained using (*X*, *y*, *c*).

### Framework

**Fig. 2:**
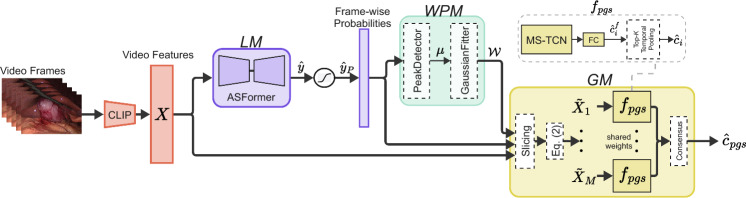
Overview of STC-Net: A frozen CLIP encoder extracts frame features $X \in \mathbb {R}^{T \times D}$. The Localization Module (LM) predicts frame-wise probabilities $\hat{y}_P$, which the Window Proposal Module (WPM) converts into candidate windows $\mathcal {W}$. The Grading Module (GM) integrates *X*, $\hat{y}_P$, and $\mathcal {W}$ to predict the final grade $\hat{c}_{\text {pgs}}$

As shown in Fig. 2, given a surgical video encoded into features *X*, STC-Net first applies the LM to generate frame-wise scores indicating the likelihood of each frame corresponding to the target timestamp. These scores are then passed to the WPM, which proposes candidate temporal segments (start and end timestamps) most informative for the estimation of PGS. For each proposal, the corresponding feature slice of *X* is extracted and processed independently by the GM, which aggregates the individual predictions to yield the final PGS estimate.

**Feature extraction:** To obtain frame-level visual representations *X*, we leverage the visual encoder of CLIP [19] with a ViT-B/16 backbone. The encoder is pre-trained in a CLIP-style manner on a large-scale in-house dataset of LC videos using text prompts describing surgical phases and anatomical structures, using full fine-tuning. This pretraining dataset is entirely disjoint from the dataset used in this paper, and thus from all training/validation/test splits.

**Localization Module:** The LM takes CLIP features $X \in \mathbb {R}^{T\times D}$ as input and outputs frame-level scores $\hat{y} \in \mathbb {R}^T$. We implement LM with ASFormer [20], which is well suited for modeling long-range temporal dependencies and has also been successfully used in surgical action recognition settings [21]. Frame-wise probabilities can be obtained by applying a sigmoid activation to $\hat{y}$, resulting in $\hat{y}_{p} \in \left[ 0,1 \right] ^{T}$, where each value indicates the estimated probability that the corresponding frame matches the target timestamp. The predicted target timestamp is $\hat{t} = \arg \max _{i \in \{0, \dots , T-1\}} \hat{y}_i$.

**Window Proposal Module:** The WPM aims to extract temporally localized segments around key frames predicted as informative for grading by the LM, serving as focused input for the downstream PGS classification task. To identify candidate temporal regions, we apply a peak detection function $\textrm{PeakDetector}(\cdot )$ to $\hat{y}_P$, which identifies *M* local maxima using a peak probability threshold (signal’s peak height) $\theta _{prob}$ and a minimum peak distance threshold between consecutive peaks $\theta _{dist}$. The resulting peak indices $\mu = [\mu _1, \dots , \mu _M]$ indicate frames with high confidence in being the target timestamp, and *M* denotes the number of proposals. We fit two Gaussians on either side of each peak located at $\mu _i$, estimating the standard deviations $\sigma _{l,i}$ and $\sigma _{r,i}$ for the left and right portions of the signal $\hat{y}_P$, respectively. Formally, for each side, we fit an one-dimensional Gaussian function (via nonlinear least squares) of the form:

1 $$\begin{aligned} g(\tau ) = A \cdot \exp \left( -(\tau - \mu _i)^2/2\sigma _i^2 \right) , \end{aligned}$$

where $\tau \in [0, \mu _i]$ for the left side and $\tau \in [\mu _i, T]$ for the right side of the peak, *A* denotes the amplitude, and $\sigma _i \in \{\sigma _{l,i}, \sigma _{r,i}\}$. *A* is set to 1, and we solve for $\sigma _i$. The standard deviations are used to dynamically define the window boundaries around the peak, controlled by a scale hyperparameter $N_{std}$, as $(l_i, r_i) = \mu _i \pm N_{\textrm{std}} \cdot (\sigma _{l,i}, \sigma _{r,i})$. The resulting set of proposals is denoted by $\mathcal {W} = \{(l_1, r_1), \dots , (l_M, r_M)\}$ (see Alg. 1 of Online Resource 1). Unlike fixed-length windows, our dynamic approach adapts window size based on localization confidence.

**Grading module:** The GM processes the set of candidate temporal windows identified by the WPM to make PGS predictions. For each window $(l_i, r_i)$ in $\mathcal {W}$, features $X_i$ and probabilities $\hat{y}_{Pi}$ are extracted via a slicing operation ($[l_i:r_i]$) and combined as:

2 $$\begin{aligned} \tilde{X}_i = X_i \odot \hat{y}_{P_i} + X_i, \end{aligned}$$

where ⊙ denotes element-wise multiplication. This operation amplifies regions of high confidence while preserving the original signal with the skip connection. Additionally, it maintains a direct computational graph connection between the LM and the GM, enabling gradient flow from the classification objective back to the localization.

The reweighted feature segment $\tilde{X}_i$ is then forwarded to the PGS model $f_\textrm{pgs}(\cdot )$, which produces raw classification logits $\hat{c}_i \in \mathbb {R}^{C+1}$, where the additional class at index 0 denotes background (i.e., frames deemed uninformative for PGS estimation). This process is applied to all *M* proposals, yielding a set of predictions $\{\hat{c}_1, \dots , \hat{c}_M\}$. A consensus block $\textrm{Consensus}(\cdot )$ is then applied over this set to derive the final grade prediction $\hat{c}_{\text {pgs}} \in \{1, \dots , C\}$ (see Alg. 2 of Online Resource 1).

As shown in Fig. 2, the PGS model $f_\textrm{pgs}(\cdot )$ uses MS-TCN [22] for temporal modeling within each proposal (see Online Resource 1 for a comparison to a transformer-based alternative). The reweighted segment $\tilde{X}_i$ is fed into the MS-TCN to produce frame-wise logits $\hat{c}_i^f \in \mathbb {R}^{T_i \times (C+1)}$, which are then aggregated by top-*K* pooling [15] into the proposal-level prediction $\hat{c}_i \in \mathbb {R}^{C+1}$. As for the $\textrm{Consensus}(\cdot )$ block, we select the proposal with the highest localization confidence $i^* = \arg \max _{i} \hat{y}[\mu _i]$ and predict the most confident (non-background) class, $\hat{c}_{\text {pgs}} = \arg \max _{j \in \{1, \dots , C\}} \hat{c}_{i^*, j}$.

## Experimental design

### Training Objectives

**Localization:** We employ a dual-loss strategy composed of a custom binary cross-entropy (BCE) loss and a cosine similarity loss.

The BCE loss treats the localization as frame-wise binary classification, where the label vector $y \in \{0,1\}^T$ has $y_t = 1$ at the target timestamp and 0 elsewhere. To reduce penalties on frames temporally close to the target, we define a neutral zone of size 3δ around *t*. Negative loss terms are applied only to frames outside this zone, while $\delta \in \mathbb {N}^+$ controls the tolerance. The BCE loss is defined as:

3 $$\begin{aligned} \mathcal {L}_{\textrm{bce}} = - y_{t} \log \hat{y}_{P,t} - \frac{1}{T-1} \sum \limits _{\begin{array}{c} j = 0 \\ j \notin [t - 3\delta ,\, t + 3\delta ] \end{array}}^{T - 1} (1 - y_j) \log (1 - \hat{y}_{P,j}) \end{aligned}$$

While BCE enforces sharp separation between the target and other frames, it ignores that nearby frames may contain relevant cues. To soften supervision, we introduce a cosine similarity loss that aligns the softmax-normalized predictions $\hat{y}_{\text {soft}}$ (along the temporal dimension of $\hat{y}$) with a Gaussian reference $\mathcal {N}(t,\delta ^2) \in \mathbb {R}^T$ centered at *t*, and with standard deviation of δ. We define this loss as:

4 $$\begin{aligned} \mathcal {L}_{\text {cos}} = 1 - \frac{\hat{y}_{\text {soft}} \cdot \mathcal {N}(t, \delta ^2)}{\left\| \hat{y}_{\text {soft}} \right\| _2 \cdot \left\| \mathcal {N}(t, \delta ^2) \right\| _2} \end{aligned}$$

The $\mathcal {L}_{\text {bce}}$ enforces precision at the target frame, while $\mathcal {L}_{\text {cos}}$ encourages smoother scores around it, mitigating overfitting from temporal ambiguity or annotation noise. The total localization loss is $\mathcal {L}_{\text {L}} = \mathcal {L}_{\text {bce}} + \alpha \mathcal {L}_{\text {cos}}$, where α is a weight hyperparameter.

**Grading:** Given a video-level grade label $c \in \{1,\dots ,C\}$ and a single ground-truth timestamp *t*, we assign *proposal-level pseudo-labels* to the *M* window proposals produced by the WPM. Specifically, the proposal whose center (peak) $\mu _i$ is closest to *t*, (i.e., $i^+ = \arg \min _{i} \left| \mu _i - t\right|$), is treated as the *foreground* (positive) instance and is supervised with label *c*. All remaining proposals are treated as *background* (negative) instances and are assigned the background label 0. The grading loss combines cross-entropy on the positive proposal with an average penalty encouraging negatives to be classified as background:

5 $$\begin{aligned} \mathcal {L}_{\text {G}}&= -\log \left( \frac{\exp (\hat{c}_{i^+, c})}{\sum _{j=0}^{C} \exp (\hat{c}_{i^+, j})} \right) \nonumber \\&\quad - \frac{1}{M-1} \sum _{\begin{array}{c} i=1 \\ i \ne i^+ \end{array}}^{M} \log \left( \frac{\exp (\hat{c}_{i,0})}{\sum _{j=0}^{C} \exp (\hat{c}_{i, j})} \right) \end{aligned}$$

**Training scheme:** We adopt a two-stage training scheme. In the first stage, only the LM is trained using the localization objective $\mathcal {L}_{\text {L}}$, while the GM remains frozen. This allows the LM to identify informative timestamps without interference from the grading task. After a fixed number of epochs, marked by the frozen epoch $E_\textrm{frozen}$, we switch to joint training, with the total loss defined as $\mathcal {L} = \mathcal {L}_G + \beta \mathcal {L}_L$, where β controls the contribution of the localization loss.

**Inference:** Note that the timestamp *t* is available only during training for weak supervision and proposal pseudo-labeling. At test time, no timestamps are used. Given a full-length video, the LM predicts frame-wise relevance scores $\hat{y}$, the WPM generates proposals around predicted peaks $\{\mu _i\}$, and we select the proposal with the highest localization confidence to produce the final PGS prediction, as defined in “Framework” section.

### Dataset and evaluation

We evaluate STC-Net on a multi-center private dataset of 1,859 LC videos, collected from 30 institutions across 10 countries, each annotated with a PGS grade (1–5) and a single timestamp. Each video was independently annotated by at least two trained annotators (trained and assessed against expert surgeon-graded reference cases), and the final label is based on agreement between at least two annotators. A held-out test set of 190 videos is used for evaluation. Train/validation/test splits are constructed using stratified sampling by PGS grade to preserve the class distribution across splits (see Online Resource 1). For evaluation, we use the following metrics: For PGS classification, we use accuracy, precision, recall, F1-score, and average distance (AD), defined as the mean absolute error between ground-truth and predicted grades, and for localization, we use the mean absolute error (MAE) between predicted and ground-truth timestamps.

### Implementation details

STC-Net is trained with Adam for 100 epochs ($lr=1 \times 10^{-5}$, batch size 1, $E_{\text {frozen}}=8$). Videos are downsampled to 1 Hz, and *D* is 768. The ASFormer uses an encoder and a 3-stage decoder (10 layers each, feature size 64). The MS-TCN uses 1 stage with 2 layers, feature size 64, dropout 0.2. K=8 for top-*K* pooling. Peaks are detected using SciPy’s find_peaks with $\theta _\textrm{prob}=0.5$, and $\theta _\textrm{dist}=400$, while Gaussians are fitted with SciPy’s curve_fit. Other parameters are set as δ=50, $\alpha =\beta =1$, and $N_\textrm{std}=2$. STC-Net is implemented in PyTorch v2.4 and trained on an NVIDIA RTX A6000 (48 GB). More hyperparameter details and analysis can be found in the Online Resource 1.

**Table 1:** Baseline performance on the PGS classification task

Model Input	Loc. Supervision	Method	Accuracy (↑)	Precision (↑)	Recall (↑)	F1-Score (↑)	AD (↓)	MAE (↓)
Full Videos	None	*Full	46.67 (±4.38)	48.19 (±2.91)	50.19 (±3.58)	46.82(±4.19)	0.73 (±0.07)	NA
Trimmed Clips	NA (manually clipped, ideal localization)	^†^Trimmed (20s)	61.58 (±0.91)	61.20 (±0.83)	61.72 (±1.66)	60.86 (±1.09)	0.46 (±0.01)	NA
		^†^Trimmed (60s)	60.70 (±1.99)	60.69 (±1.79)	61.56 (±1.79)	60.65 (±1.56)	0.50 (±0.03)	
		^†^Trimmed (120s)	58.25 (±1.99)	58.15 (±2.31)	58.32 (±1.37)	57.92 (±2.01)	0.51 (±0.01)	
		^†^Trimmed (180s)	58.24 (±2.49)	58.52 (±2.31)	57.70 (±2.46)	57.49 (±2.61)	0.53 (±0.04)	
		^†^Trimmed (240s)	56.67 (±2.65)	56.57 (±2.98)	56.21 (±2.49)	55.84 (±3.02)	0.55 (±0.03)	
Full Videos	Single timestamp	STC-Net	60.18 (±1.69)	60.70 (±2.46)	60.06 (±1.87)	59.68 (±1.51)	0.50 (±0.01)	85.38 (±10.95)

*Without and ^†^with use of target timestamps at train/test time. MAE is evaluated only for localization. Results are averaged over 3 runs

## Results and discussion

### Comparison with baselines

To the best of our knowledge, no prior work has addressed PGS estimation from full-length surgical videos that jointly perform temporal localization and grading. Therefore, to contextualize the performance of our approach, we design a set of representative baselines for comparison. The *Full* baseline processes the entire video as input, without any use of the target timestamp during training or test time. *Full* serves as a lower bound, highlighting the difficulty of learning from long videos where irrelevant content can dilute relevant information for grading. To approximate an upper bound with ideal localization, we use the *Trimmed* baseline, which uses ground-truth timestamps at test time. Videos are cropped to symmetric windows around the annotated timestamp for training and testing (see Table 1). For a fair comparison, all baselines use the same precomputed CLIP features and GM architecture as STC-Net.

As shown in Table 1, STC-Net significantly outperforms the *Full* baseline, with +13.51% and +12.86% increases in accuracy and F1-score, respectively, highlighting the importance of temporal localization for downstream PGS estimation, even when provided only through weak supervision. Also, STC-Net performs comparably to the *Trimmed* variants (20 s and 60 s), demonstrating that its weakly supervised training alone can match the performance of a model with ground-truth localization access at test time.

**Fig. 3:**
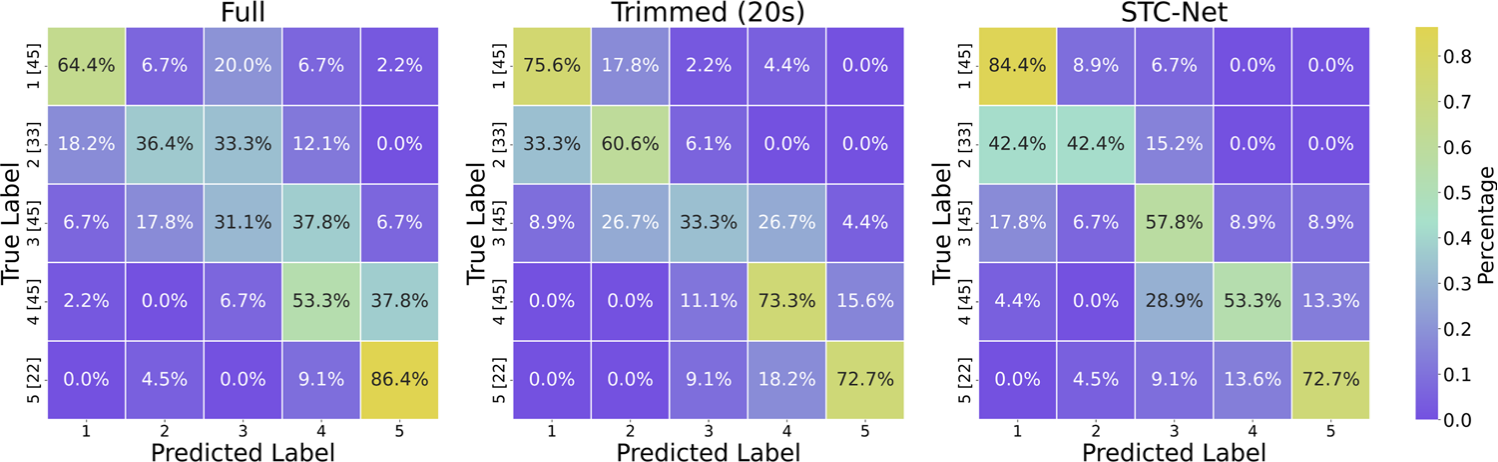
Confusion matrices for *Full*, *Trimmed (20s)*, and STC-Net (left to right) on the PGS classification task. Numbers in [.] indicate the number of videos per class

To analyze model behavior, we present confusion matrices in Fig. 3. STC-Net performs well on Grades 1 and 5, while showing some confusion among intermediate grades, particularly Grades 2 and 4. However, most misclassifications occur between adjacent classes, indicating that errors are generally mild. This aligns with the low AD of 0.5, showing that incorrect predictions remain close to the true grade. This is important in clinical practice as the model rarely confuses extreme grades, thus minimizing the risk of severely under- or overestimating disease severity. The *Trimmed* baseline (20s) handles Grades 2 and 4 slightly better, likely due to its access to ground-truth timestamps at test time. In contrast, STC-Net performs better on Grade 1 and nearly doubles accuracy on Grade 3. These results show that minimal, low-cost timestamp supervision can match methods relying on manually trimmed (ideal) localization.

Furthermore, a reason why all models tend to underperform on the intermediate classes may stem from the clinical definition of the PGS itself, where overlapping criteria [6] (*e.g.,* presence of adhesions) can blur the distinction between adjacent grades in terms of inflammation severity, whereas Grades 1 and 5 exhibit more distinct visual characteristics, making them easier to discriminate (see Online Resource 1 for more on failure modes). Finally, while STC-Net and the *Trimmed* baseline outperform the *Full* model on most classes, the latter does better on Grade 5. This suggests that for accurate Grade 5 prediction, localization, whether precise (*Trimmed*) or estimated (STC-Net), offers no benefit; instead, access to the entire video is required. Clinically, Grade 5 reflects severe, widespread inflammation that may manifest in multiple surgical phases, so global video context may be needed to capture its defining features. For example, the gallbladder may be obscured by adhesions, with additional signs of inflammation only visible after their removal.

### Ablation studies

**Analysis of the Window Proposal Module:** We assess key design choices in the WPM through experiments with the following configurations: (i) We remove the WPM entirely. Without proposal generation, the entire sequence is fed directly into the GM. Equation 2 reduces to $\tilde{X} = X \odot \hat{y}_{P} + X$, and no slicing is applied. This experiment evaluates the benefit of explicitly focusing on localized segments versus processing the full video; (ii) we replace the dynamic window generation algorithm ($\textrm{GaussianFitter}$) with predefined fixed-size windows centered around each detected peak. This configuration isolates the impact of dynamic window sizes tailored to each video. Table 2 presents the results for ablations (i) and (ii).

Ablation (i) highlights the critical role of the WPM: Removing it and feeding the full sequence into the GM lead to a −14.22% accuracy drop. This setup even underperforms the *Full* baseline (Table 1), which does not use any localization, suggesting that localization and window-level slicing should be combined to be effective. Ablation (ii) shows that dynamic window proposals with fitted Gaussians outperform fixed-size (240s) slicing by +2.11% in accuracy, indicating that variable-length windows are better suited to capturing video-specific temporal context. This gain likely stems from different videos containing varying numbers of frames that are informative for PGS estimation, something dynamic windows adapt to more effectively than fixed-size ones. Ablation (ii) also shows that even fixed-size slicing around detected peaks outperforms models without slicing (first row of Tables 1 and 2), highlighting the importance of providing the GM with focused temporal proposals (i.e., slices) rather than full sequences (except for Grade 5).

**Table 2:**
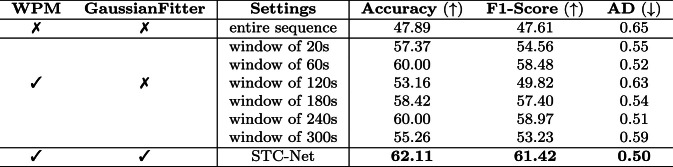
Ablation studies of the Window Proposal Module

Bold values indicate the best performance

**Table 3:**
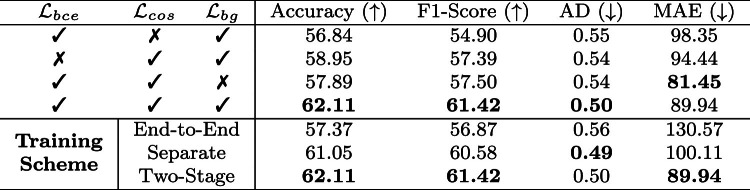
Ablating losses and training schemes

Bold values indicate the best performance

**Fig. 4:**
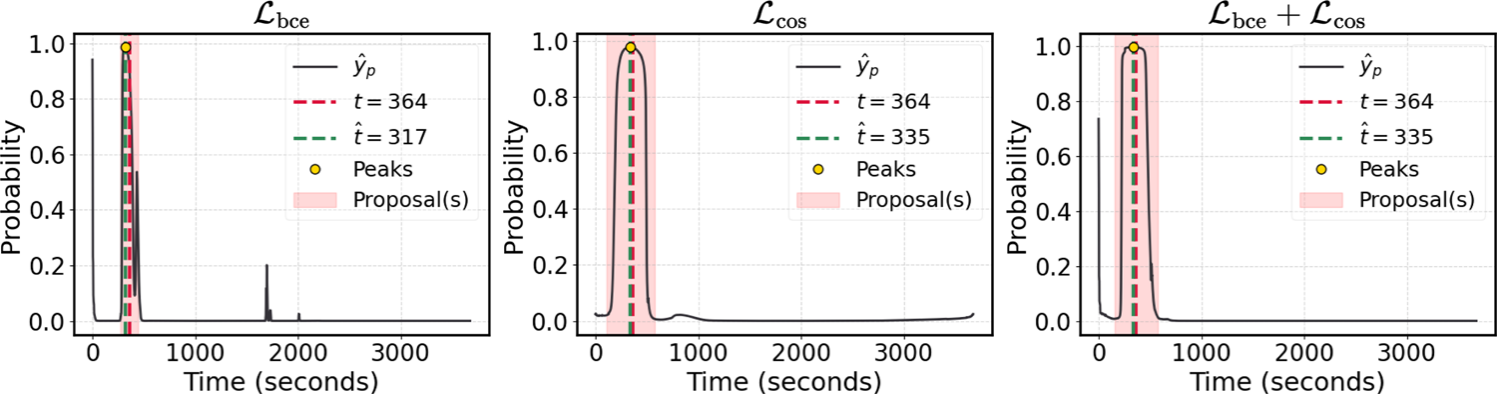
Qualitative comparison of frame-wise localization probabilities $\hat{y}_P$ under different supervision: from left to right, $\mathcal {L}_{\text {bce}}$, $\mathcal {L}_{\text {cos}}$, and $\mathcal {L}_{\text {bce}}+\mathcal {L}_{\text {cos}}$

**Table 4:** Impact of different consensus types

Consensus	Accuracy (↑)	F1-score (↑)	AD (↓)
Average	59.47	58.81	0.53
Majority vote	61.05	60.60	0.52
Highest confidence	61.58	60.86	0.51
Highest peak (proposed)	**62.11**	**61.42**	**0.50**

Bold values indicate the best performance

**Analysis of training losses and scheme:** Table 3 shows the contribution of each localization loss ($\mathcal {L}_{\text {bce}}$, $\mathcal {L}_{\text {cos}}$) and the background term of the grading loss (2nd term of Eq. 5, denoted as $\mathcal {L}_{\text {bg}}$). Removing $\mathcal {L}_{\text {bg}}$ corresponds to supervising only the positive window, without explicit background modeling.

The combination of $\mathcal {L}_{\text {bce}}$ and $\mathcal {L}_{\text {cos}}$ outperforms either loss alone. We attribute this to $\mathcal {L}_{\text {bce}}$ enforcing sharp peaks at the target timestamp without penalizing neutral frames, while $\mathcal {L}_{\text {cos}}$ provides soft supervision by encouraging a smooth Gaussian-like shape around it, thereby handling uncertainty. Interestingly, using only $\mathcal {L}_{\text {cos}}$ outperforms $\mathcal {L}_{\text {bce}}$, suggesting that distribution-based supervision is more robust to annotation noise and temporal ambiguity. As shown in Fig. 4, $\mathcal {L}_{\text {bce}}$ yields sharp peaks which can lead to proposals that miss important context, while $\mathcal {L}_{\text {cos}}$ yields smoother, wider peaks that may include redundant frames. Their combination balances these effects, producing proposals better suited for grading.

Removing the $\mathcal {L}_{\text {bg}}$ term from the grading loss results in a noticeable drop of −4.22% in grading accuracy, showing the value of explicitly modeling uninformative frames as background, which helps the model better differentiate between relevant and irrelevant temporal content. However, localization MAE improves in the absence of $\mathcal {L}_{\text {bg}}$, but this is expected as the optimization becomes more focused on the localization objectives.

We further study the impact of the training scheme (see Table 3). We compare three strategies: *End-to-End* training, where LM and GM are jointly optimized from the start; *Separate* training, where the LM is trained first and then frozen while training the GM; and our proposed *Two-Stage*. Our *Two-Stage* scheme achieves the best overall performance by first letting the LM focus on localization and then refining it with grading supervision. *End-to-End* underperforms likely because early joint optimization yields weak proposals that hurt grading. *Separate* performs better than *End-to-End*, but still worse than *Two-Stage* since the LM is never updated with grading loss.

**Impact of consensus block:** We further compare four proposal prediction aggregation strategies for the $\textrm{Consensus}(\cdot )$ block: *Average* (mean score across proposals), *Majority Vote* (most frequent class), *Highest Confidence* (class from proposal with highest class confidence), and *Highest Peak* (see Framework section). The background class is always excluded. As shown in Table 4, naive averaging yields the weakest performance, as confident predictions may be diluted by noisy ones. Although class confidence-based strategies *Majority Vote* and *Highest Confidence* yield stronger results, focusing on the most confident proposal (*Highest Peak*) proves even more effective.

## Conclusion

We presented STC-Net, the first framework to estimate surgical complexity via the PGS from full-length LC videos, trained using weak temporal supervision. STC-Net consists of a LM for timestamp prediction, a novel WPM that generates candidate segments, and a GM that classifies them and aggregates their predictions. The framework is trained with a novel combination of soft and hard temporal supervision to align localization and grading objectives, and a background-aware loss to suppress uninformative frames. Extensive experiments on a large-scale dataset demonstrate strong performance, highlighting STC-Net’s effectiveness and its potential for real-world postoperative use.

## Limitations and future work

Although our dataset is multi-center, we do not explicitly evaluate cross-center generalization of STC-Net in this work. Center-stratified evaluation is important for clinical translation and will be addressed in future work. Additionally, our method is currently designed for offline use (e.g., retrospective evaluation/training), which limits intraoperative applicability. An online variant could provide real-time estimates of surgical complexity to support intraoperative decision-making and risk assessment and will be explored in future work. Finally, to address adjacent-grade ambiguities, future work will incorporate stronger anatomical priors and uncertainty estimation to flag low-confidence predictions.

## Supplementary Information

Below is the link to the electronic supplementary material.

Supplementary file 1 (pdf 1184 KB)
