# Supplementary material for: Learning from single timestamps: complexity estimation in laparoscopic cholecystectomy
Source: Int J Comput Assist Radiol Surg. 2026 May 19;21(6):1321–9. doi: 10.1007/s11548-026-03648-6 (PMC13451357; doi:10.1007/s11548-026-03648-6)
Supplement: Supplementary file 1 [file 11548_2026_3648_MOESM1_ESM.pdf]

Online Resource 1 for IPCAI Paper ID 84: Learning from Single Timestamps: Complexity Estimation in Laparoscopic Cholecystectomy

Dimitrios Anastasiou[1,3*], Santiago Barbarisi[2], Lucy Culshaw[2], Jayna Patel[2], Evangelos B. Mazomenos[1,3], Imanol Luengo[2], Danail Stoyanov[1,2,4]

[1]UCL Hawkes Institute, University College London, London, UK.
[2]Medtronic plc., London, UK.
[3]Dept of Medical Physics & Biomedical Engineering, University College London, London, UK.
[4]Dept of Computer Science, University College London, London, UK.

*Corresponding author(s). E-mail(s): dimitrios.anastasiou.21@ucl.ac.uk;

1 Grade Examples Descriptions

In Figure 1 of the main paper, we have provided examples of different PGS grades. A more detailed description of the anatomical landmarks presented in those images is given below:

1. **Images with Grade 1:** Normal appearing gallbladders.
2. **Images with Grade 2:** Normal gallbladders with minor adhesions, mostly at the neck.
3. **Images with Grade 3:** More adhesions and signs of hyperemia (bottom image).
4. **Images with Grade 4:** Adhesions obscuring majority of the GB (top), hyperemic and distended GB (bottom).
5. **Images with Grade 5:** Hyperemic and distended GB with pus (top), inability to visualize the GB due to adhesions (bottom).

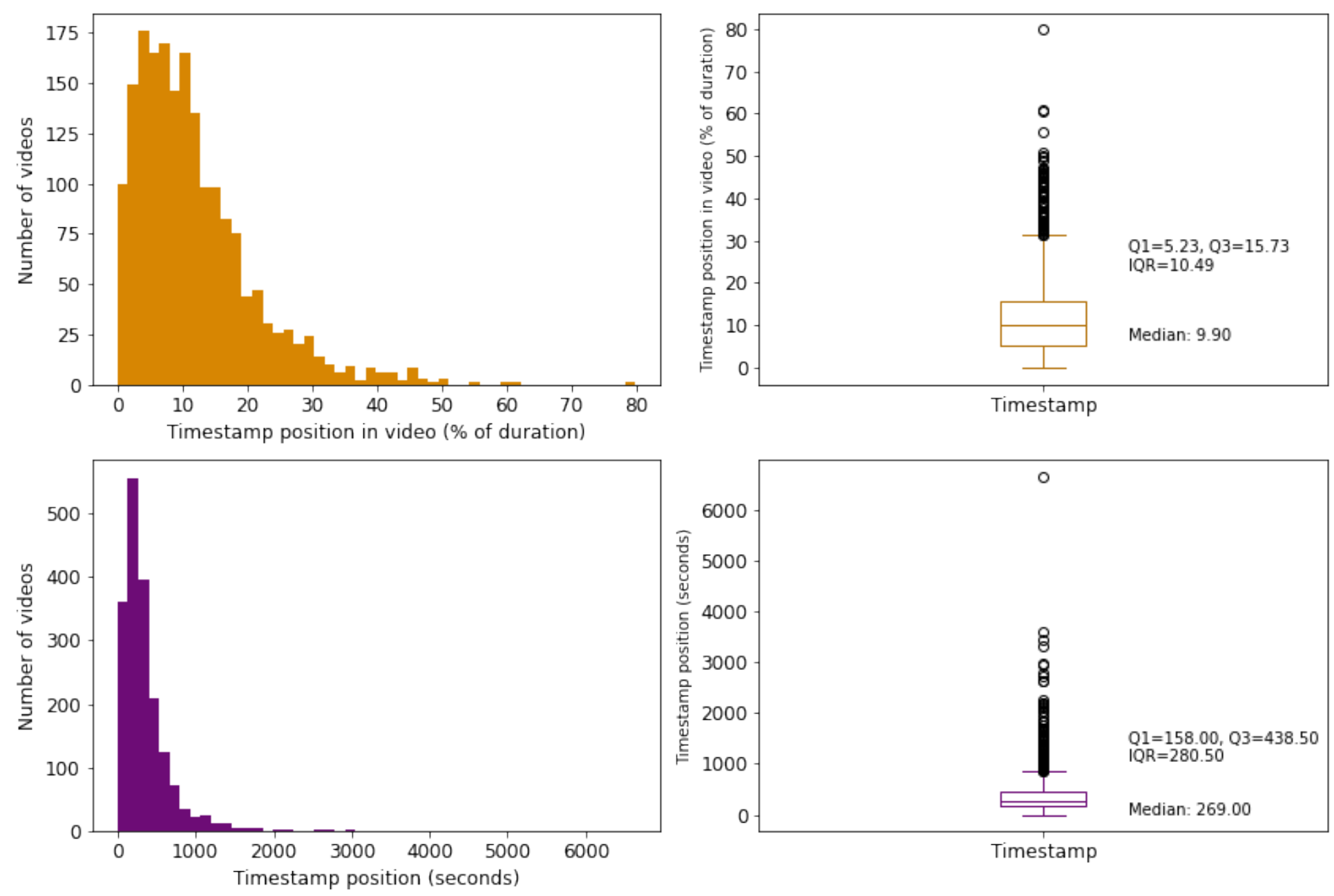

Fig. 1: Distribution of PGS timestamp annotations: **(Top row)** Distribution of the annotated timestamp location normalized by video duration ($100\% \cdot t/T$), **(Bottom row)** Distribution of the same timestamps in absolute time (seconds).

2 Dataset Analysis

To better characterize the timing of expert annotations, we add the analysis in Fig. 1. The first row reports the distribution of the timestamp location normalized by video duration ($100\% \cdot t/T$), shown as both a histogram and a box plot. The second row reports the absolute timestamp values in seconds. Since video durations vary substantially (see Fig. 2, duration in minutes), the normalized timestamp t/T is more informative. We observe that the median annotation occurs at approximately 10% of the total video duration, with some outliers beyond 30%. In absolute time, the median timestamp is 269 s (4 min 29 s), further supporting that PGS labeling typically occurs near the beginning of the procedure, with some cases going beyond that. Note that in a subset of videos, the preparation phase is substantially longer, which shifts the timestamp to a later position in the full video timeline.

3 Dataset Splits

The class distribution is: Grade 1 (443), Grade 2 (318), Grade 3 (444), Grade 4 (445), and Grade 5 (209). We construct the train/validation/test splits using stratified sampling by PGS grade, so that the class distribution in the validation and test sets is consistent with the training distribution, as shown in Table 1.

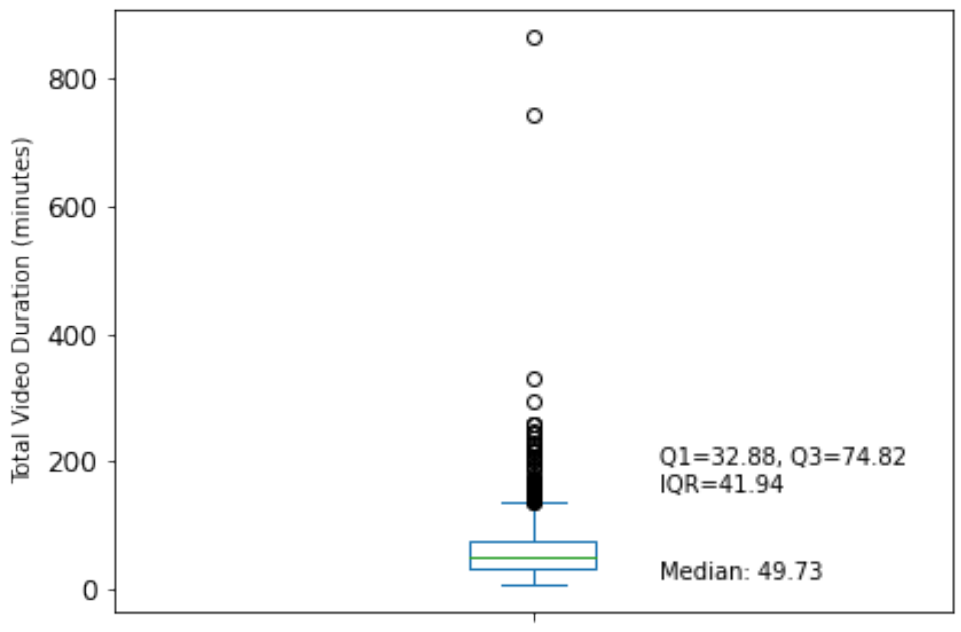

Fig. 2: Distribution of video duration in minutes.

Table 1: Number of videos per PGS grade for training/validation/test splits.

Split	Grade 1	Grade 2	Grade 3	Grade 4	Grade 5
Training Set	395	254	355	356	167
Validation Set	43	31	44	44	20
Test Set	45	33	45	45	22

4 Algorithms

A more detailed presentation of the data flow for the Window Proposal Module (WPM) and the Grading Module (GM) is provided in Algorithm 1 and Algorithm 2, respectively.

Algorithm 1: Window Proposal Module

Input: frame-level localization scores $\hat{y}_P \in \mathbb{R}^T$
Output: set of window proposal boundaries $\mathcal{W} = \{(l_1, r_1), \ldots, (l_M, r_M)\}$
$\mu \leftarrow \text{PeakDetector}(\hat{y}_P)$;
$\mathcal{W} \leftarrow \emptyset$;
for $\mu_i \in \mu$ **do**
  $\sigma_{l,i} \leftarrow \text{GaussianFitter}(\hat{y}_P[0 : \mu_i]; \mu_i)$;
  $\sigma_{r,i} \leftarrow \text{GaussianFitter}(\hat{y}_P[\mu_i : T]; \mu_i)$;
  $l_i \leftarrow \mu_i - N_{\text{std}} \cdot \sigma_{l,i}$;
  $r_i \leftarrow \mu_i + N_{\text{std}} \cdot \sigma_{r,i}$;
  $l_i \leftarrow \max(0, \lfloor l_i \rfloor)$;
  $r_i \leftarrow \min(T - 1, \lceil r_i \rceil)$;
  $\mathcal{W} \leftarrow \mathcal{W} \cup \{(l_i, r_i)\}$;

Algorithm 2: Grading Module

Input: video features $X \in \mathbb{R}^{T \times D}$, frame-level localization probabilities $\hat{y}_P \in \mathbb{R}^T$, set of window proposal boundaries $\mathcal{W} = \{(l_1, r_1), \ldots, (l_M, r_M)\}$
Output: PGS class prediction $\hat{c}_{pgs} \in \mathbb{R}^C$

$\hat{c} \leftarrow []$; `// Initialize list`
for (l_i, r_i) *in* $\mathcal{W}$ **do**
  $X_i \leftarrow X[l_i : r_i, :]$;
  $\hat{y}_{Pi} \leftarrow \hat{y}_P[l_i : r_i]$;
  $\tilde{X}_i \leftarrow X_i \odot \hat{y}_{Pi} + X_i$;
  $\hat{c}_i \leftarrow f_{pgs}(\tilde{X}_i)$; `//` $\hat{c}_i \in \mathbb{R}^{C+1}$
  $\hat{c} \leftarrow \hat{c}.\text{append}(\hat{c}_i)$;
$\hat{c}_{pgs} \leftarrow \text{Consensus}(\hat{c})$; `//` $\hat{c}_{pgs} \in \{1, \ldots, C\}$

Table 2: Comparison against frozen CLIP encoder.

Method	Accuracy (↑)	F1-Score (↑)	AD (↓)
Full (frozen CLIP)	35.26	32.42	1.11
STC-Net (frozen CLIP)	48.42	48.89	0.76
STC-Net (ours)	**62.11**	**61.42**	**0.50**

Table 3: Comparison against SF-Net [1].

Method	**Accuracy** (↑)	**F1-Score** (↑)	**AD** (↓)
SF-Net	59.47	59.66	0.54
STC-Net	**62.11**	**61.42**	**0.50**

5 Additional Experiments

5.1 CLIP Encoder

We also include an ablation with a frozen CLIP [2] visual encoder (*i.e.,* the original CLIP weights) versus our in-house LC-pretrained encoder in Table 2, showing that domain-specific pretraining yields a significant performance improvement. To further contextualize the frozen-CLIP setting, we additionally report the *Full* baseline (see the main paper Section 4.1 for definition) using frozen CLIP features, demonstrating that our framework consistently outperforms full-video grading (no localization) regardless of the feature extractor.

5.2 Additional Baselines

We also provide a comparison against SF-Net [1], a standard point-supervision baseline. We tuned SF-Net for grading-related performance (as STC-Net) and used the same fine-tuned CLIP features as in our framework for a fair comparison. As shown in

Table 4: Grading Module (GM) backbone comparison.

GM backbone	**Accuracy** (↑)	**F1-Score** (↑)	**AD** (↓)
ASFormer	54.21	50.12	0.58
MS-TCN	**62.11**	**61.42**	**0.50**

Table 5: Effect of the peak probability threshold hyperparameter θ_{prob}.

θ_{prob}	**Accuracy** (↑)	**F1-Score** (↑)	**AD** (↓)
0.3	60.00	59.03	0.51
0.5	**62.11**	**61.42**	**0.50**
0.7	59.47	57.78	0.55

Table 6: Effect of the peak distance threshold hyperparameter θ_{dist}.

θ_{dist}	**Accuracy** (↑)	**F1-Score** (↑)	**AD** (↓)
300	57.37	56.34	0.55
400	**62.11**	**61.42**	0.50
800	61.58	60.81	**0.49**
1000	57.89	56.55	0.54

Table 3, we observe that STC-Net outperforms SF-Net by 2.24% in accuracy, 1.76% in F1-score, and 0.04 in AD, indicating that our design is better suited for PGS estimation.

Note that, point-supervision methods, such as SF-Net [1], are developed for weakly supervised temporal action localization. They typically assume videos contain multiple action instances with action categories, and they are optimized for boundary-sensitive localization metrics such as mAP at different IoU thresholds that reflect how accurately the start/end times of action instances are recovered. Our problem differs substantially as each (entire) video is associated with a single ordinal label (PGS 1–5) reflecting inflammation severity, and the timestamp supervision denotes a representative grading moment rather than an action instance.

5.3 Grading Module Backbone

To justify the choice of MS-TCN in the Grading Module, we compare it against a transformer-based temporal model used as the grading backbone. Specifically, we replace MS-TCN with ASFormer [3] while keeping all other components unchanged. As shown in Table 4, MS-TCN achieves better grading performance than the transformer alternative. We hypothesize that the transformer is more prone to overfitting in this setting, which can lead to poorer generalization. Since the Grading Module

Table 7: Effect of the scale hyperparameter N_{std} on model performance.

N_{std}	**Accuracy** (↑)	**F1-Score** (↑)	**AD** (↓)
1	56.32	54.56	0.55
2	**62.11**	**61.42**	**0.50**
3	54.74	52.32	0.60

Table 8: Effect of the hyperparameter δ of $\mathcal{L}_{\text{L}}$ on model performance.

δ	**Accuracy** (↑)	**F1-Score** (↑)	**AD** (↓)	**MAE** (↓)
10	58.42	56.24	0.54	103.95
50	**62.11**	**61.42**	**0.50**	**89.94**
100	53.16	50.53	0.63	97.04

operates on window proposals that are relatively short to medium in length (approximately 400 frames on average), MS-TCN appears to be a more suitable and robust temporal aggregator for such sequences.

5.4 Hyperparameters

For the model hyperparameters, we followed the original implementations of MS-TCN [4] and ASFormer [3]. For top-k temporal pooling, we adopted the setup from [1]. The weighting coefficients α and β were both set to 1, without further tuning. All remaining hyperparameters were determined heuristically, with the results reported for PeakDetector($\cdot$) threshold hyperparameters, as well as N_{std} and δ as shown below.
Impact of PeakDetector($\cdot$) **hyperparameters:** We select peaks whose probability exceeds a certain threshold. We evaluated the values of $\{0.3, 0.5, 0.7\}$ and found that 0.5 performed best in our validation experiments. Intuitively, a higher threshold reduces the number of proposals and can miss relevant regions, whereas a lower threshold produces many proposals that can introduce noise for the downstream grading stage. We also enforce a minimum distance between detected peaks to avoid redundant proposals. We evaluated minimum distances in $\{300, 400, 800, 1000\}$ frames and selected 400 based on validation performance. Results for both hyperparameters are shown in Table 5 and Table 6.
Impact of hyperparameter N_{std}: We study the impact of the scale hyperparameter N_{std} used in the Window Proposal Module. The results are presented in Table 7, showing optimal performance with $N_{\text{std}} = 2$. Small values of N_{std} may lead to windows that are too narrow, potentially missing important contextual information, especially when localization is not accurate; conversely, large values may result in proposals that include excessive redundant information.
Impact of hyperparameter δ: Additionally, we study the effect of the scale hyperparameter δ used in the localization loss $\mathcal{L}_{\text{L}}$ (see Table 8). Smaller values of δ lead to fewer frames being treated as positives, thus increasing the number of negatives, and

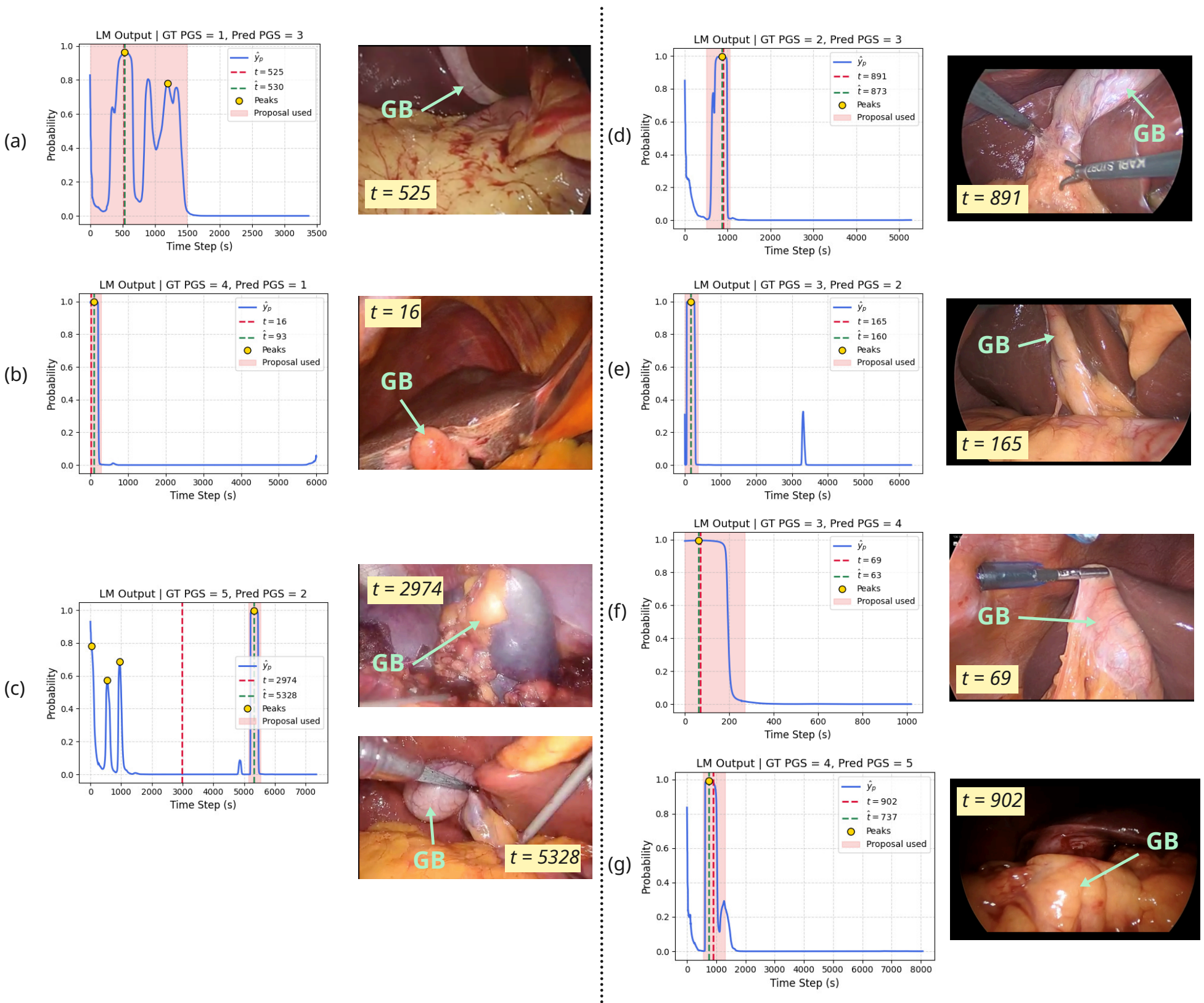

Fig. 3: Examples of misclassifications on the test set, grouped into larger errors (a–c) and adjacent-grade confusions (d–g). We show the LM output over time, the ground-truth timestamp t (red dashed line), the predicted timestamp $\hat{t}$ (green dashed line), detected peaks (markers), and the selected window proposal used for grading (shaded region). The image on the right of each panel shows a snapshot at the ground-truth timestamp; in (c), we also show a frame at the predicted timestamp.

vice versa. Our experimentation showed that a setting of $\delta = 50$ achieves the best performance balancing the ratio of positive to negative frames.

6 Failure Modes

We categorize the failure cases into two groups, as shown in Fig. 3: (a)–(c) illustrate larger grading errors, while (d)–(g) show milder (adjacent-grade) errors. Each example visualizes the localization output (with ground-truth and predicted timestamps), the selected proposal used for grading, and a representative frame.

Larger errors (a–c). In (a) (GT: 1, Pred: 3), the selected proposal is relatively wide and the Localization Module produces multiple peaks, resulting in a window that extends beyond the grading-relevant inspection moment ($t = 525$). This can

introduce confounding context (*e.g.,* later dissection and blood), biasing the grading toward higher severity. In (b) (GT: 4, Pred: 1), the proposal is narrow and may not capture sufficient inspection information, leading to high uncertainty (predicted entropy of 1.27) and an underestimation of severity, highlighting that missed context within the proposal may negatively impact grading even when some inflammatory cues are present. Finally, (c) (GT: 5, Pred: 2) is a representative localization error case: the predicted timestamp is substantially later than the ground-truth ($t = 2974$ vs $t = 5328$), yielding a proposal after partial dissection/cleaning. As a result, the gallbladder appears less inflamed in the selected window, leading to underestimation.

Milder errors (d–g). Examples (d) and (e) show Grade 2↔3 confusions. This is clinically plausible because adjacent PGS grades share overlapping criteria (*e.g.,* amount and location of adhesions) [1], and borderline cases can be difficult to separate even for experts. Examples (f) and (g) illustrate overestimation by one grade when adhesion coverage is large. Since extensive adhesions (*e.g.,* buried gallbladder) are strongly associated with higher grades (particularly 4–5), the model can be biased towards those grades.

Clinical implications. The main implication of larger grading errors is incorrect case stratification (over- or under-estimating inflammation severity), which could affect postoperative audit, training, and retrospective analyses. Importantly, such large errors are relatively rare in our evaluation (consistent with the low Average Distance and the confusion matrix results), while most errors occur between adjacent grades and are therefore typically less consequential.

References

[1] F. Ma, L. Zhu, Y. Yang, et al., Sf-net: Single-frame supervision for temporal action localization. ECCV pp. 415–431 (2020)

[2] A. Radford, J.W. Kim, C. Hallacy, et al., *Learning transferable visual models from natural language supervision*, in *ICML* (2021), pp. 8748–8763

[3] F. Yi, H. Wen, T. Jiang, Asformer: Transformer for action segmentation. BMVC (2021)

[4] Y.A. Farha, J. Gall, Ms-tcn: Multi-stage temporal convolutional network for action segmentation. CVPR pp. 3575–3584 (2019)
